# The Impact of *Escherichia coli* Probiotic Strain O83:K24:H31 on the Maturation of Dendritic Cells and Immunoregulatory Functions In Vitro and In Vivo

**DOI:** 10.3390/cells11101624

**Published:** 2022-05-12

**Authors:** Lenka Súkeníková, Viktor Černý, Jan Věcek, Petra Petrásková, Olga Novotná, Šimon Vobruba, Tereza Michalčíková, Jan Procházka, Libuše Kolářová, Ludmila Prokešová, Jiří Hrdý

**Affiliations:** 1 First Faculty of Medicine, Charles University and General University Hospital, 121 08 Prague, Czech Republic; lenka.sukenikova@lf1.cuni.cz (L.S.); viktor.cerny@lf1.cuni.cz (V.Č.); jan.vecek@lf1.cuni.cz (J.V.); petra.petraskova@lf1.cuni.cz (P.P.); olga.novotna@lf1.cuni.cz (O.N.); libuse.kolarova@lf1.cuni.cz (L.K.); lprok@lf1.cuni.cz (L.P.); 2 Faculty of Science, Charles University, 128 00 Prague, Czech Republic; 3 Czech Academy of Sciences, 142 20 Prague, Czech Republic; simon.vobruba@gmail.com; 4 Czech Centre for Phenogenomics, Institute of Molecular Genetics, Czech Academy of Sciences, 252 50 Vestec, Czech Republic; tereza.michalcikova@img.cas.cz (T.M.); jan.prochazka@img.cas.cz (J.P.)

**Keywords:** probiotic, *E. coli* O83:K24:H31, luciferase, dendritic cell, IL-10, indol amine 2,3 dioxygenase, early postnatal probiotic administration

## Abstract

Early postnatal events are important for the development of the neonatal immune system. Harboring the pioneering microorganisms forming the microbiota of the neonatal gastrointestinal tract is important for priming the immune system, as well as inducing appropriate tolerance to the relatively innocuous environmental antigens and compounds of normal healthy microbiota. Early postnatal supplementation of suitable, safe probiotics could accelerate this process. In the current study, the immunomodulatory capacity of the probiotic strain of *Escherichia coli* O83:K24:H31 (EcO83) was characterized in vitro and in vivo. We compared the capacity of EcO83 with and without hemolytic activity on selected immune characteristics in vitro as determined by flow cytometry and quantitative real-time PCR. Both strains with and without hemolytic activity exerted comparable capacity on the maturation of dendritic cells while preserving the induction of interleukin 10 (*Il10*) expression in dendritic cells and T cells cocultured with EcO83 primed dendritic cells. Early postnatal supplementation with EcO83 led to massive but transient colonization of the neonatal gastrointestinal tract, as detected by in vivo bioimaging. Early postnatal EcO83 administration promoted gut barrier function by increasing the expression of claudin and occludin and the expression of *Il10*. Early postnatal EcO83 application promotes maturation of the neonatal immune system and promotes immunoregulatory and gut barrier functions.

## 1. Introduction

Newborns are considered to be relatively sterile and, during labor, they encounter microbes in the birth canal for the first time. Recently, it was shown that, even during prenatal life, the fetus can interact with live microbes, suggesting that the priming of the immune system occurs prenatally [1,2,3]. However, some studies were not able to detect placental microbiota [4,5], highlighting that further studies are needed for the clarification of the presence of live microbes in the placenta and their role in the activation of neonatal immune system. In addition to that, the possibility of the contamination of biological material during sampling, together with the contamination of reagents used for bacterial DNA extraction, have been suggested [6]. Regardless of the possible role of placental microbiota in the priming of the fetal immune system, perinatal events, including the mode of delivery, affect the development of the neonatal immune system and the risk of generating immune-related disorders [7].

Dysbiosis (altered microbiota composition or function) in early postnatal life is associated with delayed immune system maturation, predisposing these individuals to the development of immune-mediated disorders such as allergies [8]. Microbes coming from the vaginal microbiota are able to metabolize the oligosaccharides from maternal milk, thus providing the neonate with an additional source of energy [9]. Therefore, it is not surprising that newborns delivered by Caesarean section have distinct microbiota compositions compared to neonates born vaginally [10]. The main difference in the microbiota composition between children delivered by Caesarean section and those delivered vaginally can be found in the delayed presence of *Bacteriodetes*, a lower proportion of bifidobacteria and *Bacteriodetes,* and generally lower microbial diversity [11]. On the other hand, an elevated proportion of *Klebsiella* has been documented in the gut microbiota of children delivered by Caesarean section [12,13]. Changes in gut microbiota during the course of pregnancy were observed [14] to lead to the detection of different microbiota compositions in women delivering children at term or preterm [9,10,15]. On the contrary, another study determined that no difference in microbiota between women who delivered children at term or preterm was detected [16], challenging the association of microbiota with preterm deliveries.

Probiotic supplementation seems to be an easy and rational way to correct for dysbiosis and to promote or restore mutual homeostatic interactions between microbiota and the host immune system. The introduction of probiotics, prebiotics, or synbiotics to humans and animals can be beneficial for both intestinal microbiota and host immune systems [17,18]. The capacity of probiotics to promote immunoregulatory functions has been documented [19,20,21,22,23].

The impaired immunoregulatory capacities of the immune system have been associated with a broad spectrum of immune-mediated disorders (e.g., inflammatory bowel diseases, allergies, autoimmune diseases, etc.) [24,25]. Dendritic cells (DC) are known to be a key population inducing and shaping immune responses, including the immunoregulatory response [26,27]. Other important populations with regulatory capacity are regulatory T cells (Treg), known for their capacity to produce immunoregulatory cytokines interleukin 10 (IL-10), transforming growth factor beta (TGF-β), and IL-35, as highlighted in several studies [28,29,30]. Interestingly, the capacity of distinct probiotic strains or their compounds to promote immunoregulatory capacity has also been observed [31,32,33,34]. 

In the current study, we tried to elucidate the potential modes of action of the possible beneficial effects of early postnatal probiotic supplementation on immune system maturation and the setting of immunoregulatory functions. Early postnatal probiotic supplementation can have more profound effects because of the yet undeveloped gut microbiota in neonates competing for the sources of nutrients. In addition to that, the neonatal immune system is not fully matured, suggesting broader possibilities for probiotics to shape immune responses. In our study, the probiotic strain *Escherichia coli* O83:K24:H31 (EcO83), known to lower allergy incidence and promote IL-10 production [35,36,37,38], was used. The probiotic strain EcO83 included in Colinfant Newborn was reported to exert a mild hemolytic capacity [39]. Therefore, the strain EcO83 without hemolytic capacity could represent a safer option. The immunomodulatory capacity of EcO83 with and without hemolysin has been compared in vitro. Importantly, the presence of hemolysin could be associated with the higher fitness of this particular strain of EcO83 by promoting its good colonization capacity [40]. In this study, the immunomodulatory capacity of EcO83 was tested both in vitro and in vivo. 

## 2. Materials and Methods

### 2.1. Bacteria Preparation

Probiotic strain of *Escherichia coli* O83:K24:H31 (EcO83) present in probiotic vaccine called Colinfant Newborn produced by company Dyntec was grown in Luria Bertani broth. For in vitro stimulation, bacterial suspension was diluted by phosphate buffered saline (PBS) containing 25% glycerol (*v*/*v*) to McFarland 3 (approximately 2 × 10^9^ colony forming units (CFU)/mL). For in vivo administration, bacterial suspension was washed and diluted in gavage buffer to 5 × 10^9^ CFU/mL (one dose contained 10^9^ CFU in 200 μL of gavage buffer) for adults and 5 × 10^10^ CFU/mL (one dose contained 10^9^ CFU in 20 μL) for pups. To detect the colonization capacity of probiotic bacteria, plasmid-expressing luciferase with resistance to ampicillin (pAKlux2 was a gift from Attila Karsi (Addgene plasmid # 14080; http://n2t.net/addgene:14080, accessed on 6 May 2022; RRID: Addgene_14080), Addgene Watertown, MA, USA) was inserted into EcO83 by electroporation. Strain of EcO83 without hemolysin (HLY−) was kindly provided by prof. Peter Šebo [40]. 

### 2.2. Mice

BALB/c mice were used to study the impact of early postnatal probiotic supplementation on maturation of the immune system and tracking the colonization capacity of EcO83. Pups were colonized by EcO83 within 24 h after delivery with the first dose followed by another four doses orally on four consecutive days (five doses in total, 10^9^ CFU of EcO83 in 20 μL per one dose). In experiments using neonatal mice, both females and males were included in the study. Adult mice (9-week-old female mice BALB/c) received five consecutive doses in the following five days (10^9^ CFU of EcO83 in 200 μL of gavage buffer) intragastrically. Gavage buffer was composed of PBS containing 200 mM NaHCO_3_ and 2% glucose. Mice were kept in a specific-pathogen-free (SPF) animal facility of First Faculty of Medicine, Charles University and Czech Centre for Phenogenomics at the Institute of Molecular Genetics with 12 h dark/light cycle. Mice had ad libitum access to regular chow (ST-1 purchased from Velaz, Prague, Czech Republic) and water. The animal study protocol was approved by the Institutional Review Board of Ministry of Education, Youth and Sports (protocol code MSMT-17298/2021-4 and MSMT-17296/2021-5). For tracking the persistence of EcO83 in neonatal gastrointestinal tract by bioimaging, 10^9^ CFU of EcO83 with luciferase in 20 μL was administered to newborn mice orally. At day 10 after EcO83 supplementation, parts of the gastrointestinal tracts were collected and stored in RNA for further gene expression analyses later. Remaining parts of small intestine were collected for cell isolation and characterization of cellular subsets by flow cytometry. Mesenteric lymph nodes (MLN) were collected. Cell suspensions were prepared from MLN for flow cytometry analyses.

### 2.3. Preparation and Stimulation of Bone Marrow–Derived Dendritic Cells

Bone marrow–derived dendritic cells (BMDC) were prepared as described previously [41,42]. Briefly, progenitor cells were flushed from femur and tibia from 8-week-old female BALB/c mice followed by 10-day cultivation in the presence of recombinant murine growth factors IL-4 (cat. no. 2014-14) and GM-CSF (cat. no. 315-03) 10 μg/mL (all Peprotech, Lodon, UK). At day 10, cells were harvested and seeded in 12-well plates (2 × 10^6^ cells/well) for an additional three days, followed by stimulation with LPS from *Escherichia coli* O26:B6 (1 μg/mL; Sigma (now Merck, Darmstadt, Germany), cat. no. L2654) and EcO83 using different ratios ranging from 1000 bacterial cells to 1 bacteria:10 BMDC. To determine the optimal time point for gene expression analyses, different time intervals were tested (0.5; 1; 4 and 24 h). After stimulation, cells were collected, spun, and total RNA was extracted for gene expression analyses using quantitative real-time PCR. 

### 2.4. Coculture of EcO83-Primed BMDC with Naïve CD4^+^ T Cells

EcO83-stimulated BMDC were cocultured with naïve CD4^+^ T cells, as described previously [41,43]. Briefly, EcO83-stimulated BMDC were cocultured with CD4^+^ T cells isolated from spleen in ratio of 1 BMDC to 10 CD4^+^ T cells. Total RNA was extracted after 7 days of coculture of CD4^+^ T cells with EcO83-primed BMDC and gene expression of selected cytokines was determined by quantitative real-time PCR.

### 2.5. Flow Cytometry

The capacity of EcO83 to promote maturation of BMDC was followed by flow cytometry. After 24 h stimulation, BMDC were spun, stained by fluorochromes conjugated antibodies pointing to maturation of BMDC (CD11c PE-Cy7 (clone N417); CD80 FITC (clone 16-10A1); CD86 PE-Cy5 (clone GL1); and MHCII PE (clone M5/11.15.1.) (all eBioscience, San Diego, CA, USA) and analyzed by flow cytometry using BD FACS Canto II (Becton Dickinson, Franklin Lakes, NJ, USA). To characterize the impact of early postnatal EcO83 supplementation on maturation of immune cells, cells from lamina propria from different parts of gastrointestinal tract were isolated using enzymatic digestion by DNaseI and collagenase D. CD45^+^ cells were isolated using CD45 isolation kit (StemCells, Vancouver, BC, Canada). Isolated cells from lamina propria were characterized by flow cytometry. Pan DC isolation kit was used to obtain dendritic cells from intestine of control mice and EcO83 supplemented mice (cat. no. 130-100875; Miltenyi Biotec, Bergisch Gladbach, Germany). CD4^+^ T cells were isolated from spleen using CD4^+^ T cells isolation kit (cat. no. 130-098-248, Miltenyi Biotec). Analyses of intracellular cytokines in CD4^+^ T cells was performed using BD Transcription Factor Buffer Set (cat. no. 562574, Becton Dickinson, Franklin Lakes, NJ, USA) and antibodies directed against CD4 FITC (clone GK1.5, BioLegend, San Diego, CA, USA) or CD4 APC-Cy7 (clone GK1.5, BioLegend, San Diego, CA, USA), IFN-γ PE-Cy7 (clone XMG1.2, Sony Biotechnology Inc, San Jose, CA, USA), IL-10 PE-Cy7 (clone JES5-16E3, BioLegend, San Diego, CA, USA), IL-13 PE (clone eBio13A, eBiosciences, San Diego, CA, USA), IL-17A APC-Cy7 (TC11-18H10.1, BioLegend, San Diego, CA, USA) and FoxP3 PE (clone NRRF-30, eBiosciences, San Diego, CA, USA) according to the manufacturer recommendation. Flow cytometry data were analyzed using FlowJo (TreeStar, Ashland, OR, USA). 

### 2.6. Gene Expression Analyses

Total RNA from tissue or cells was extracted using RNeasy Mini Kit (Qiagen, Hilden, Germany). Gene expression of cytokines, tight junction proteins, inducible nitric oxice (NO) synthase [44], and indol-amine 2,3 dioxygenase (IDO) was determined as described previously [45]. Briefly, extracted RNA was reverse transcribed using High Capacity RNA to cDNA kit (ThermoFisher, Waltham, MA, USA). TaqMan gene expression assays were used to quantify the impact of EcO83 on expression of target genes in tissue and cells (BMDC and CD4^+^ T cells). List of TaqMan assays is provided in Table 1. Relative quantification of gene expression was related to the level of gene expression of beta-actin, which was used as a reference gene (housekeeping gene).

### 2.7. ELISA

Production of cytokines on protein levels in in vitro and in vivo experiments was detected by ELISA. Concentration of cytokines in cell culture supernatants was tested by ELISA using reagents purchased from R&D system (Minneapolis, MN, USA). For detection of IL-2, IL-4, and IL-10, duosets were used (cat. no. DY402, DY404-05, and DY417, respectively). For determination of IL-1β, IL12p70, IL-13, interferon gamma (IFN-γ), and tumor necrosis factor alpha (TNF-α), combinations of capture and biotinylated antibodies together with recombinant standard proteins were used (for IL-1β: monoclonal capture antibody MAB401, recombinant standard protein for creation of calibration curve 401-ML, biotinylated antibody BAF401; IL-12p70: MAB419, 419-ML, BAF419; IL-13: MAB413, 413-ML, BAF413; IFN-γ: MAB785, 485-ML, BAF485; TNF-α: AF-410-NA, 410MT, BAF410). All samples were assayed in doublets and concentration was read from a calibration curve. Standard calibration curve was calculated and data were analyzed using software KIM (Schoeller Instruments, Prague, Czech Republic).

### 2.8. In Vivo Bioluminiscence Imaging

To track the capacity of EcO83 to colonize gastrointestinal tract of newborn mice, EcO83-expressing luciferase was used. The bioluminescence was detected after luciferin Xenolight (Perkin Elmer, Waltham, MA, USA) injection (150 mg luciferin/kg body weight) followed by LagoX (Spectral instruments) in vivo whole body imaging system after 5 min post injection. The bioluminescence signal was evaluated on Aura software (Spectral instruments, Tucson, AZ, USA), where quantum yield was quantified in photons/s/cm^2^/sr.

### 2.9. Statistical Analyses

GraphPad Prism software was used for graphical and statistical evaluation and processing of data obtained. Statistical significance was determined using non-parametric one-way analysis of variance followed by Dunn multiple comparison post hoc test and two-way ANOVA with Bonferroni post hoc tests (GraphPad Prism software, San Diego, CA, USA). Data with *p* values ≤ 0.05 were considered to be statistically significant. 

## 3. Results

### 3.1. The Impact of EcO83 on BMDC Maturation

**Figure 1:**
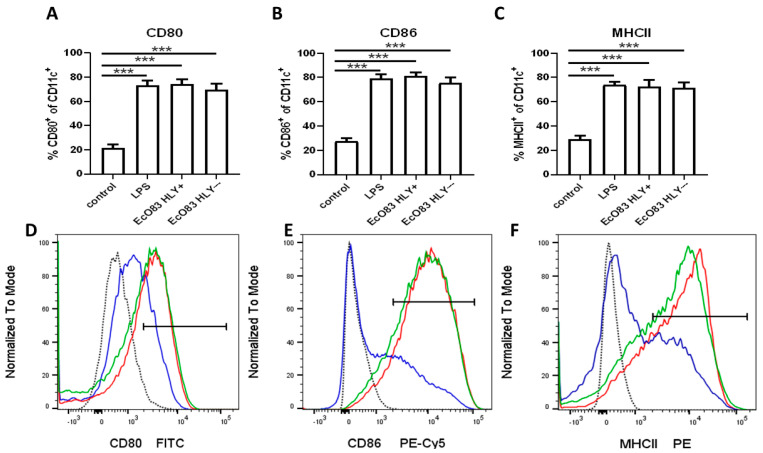
The capacity of probiotic strains of *E. coli* O83:K24:H31 (EcO83), either with hemolysin (HLY+) or without hemolysin (HLY−) activity to promote maturation of bone marrow–derived cells (BMDC) evaluated by flow cytometry. After 24 h of cocultivation of BMDC with EcO83 HLY+, EcO83 HLY−, or lipopolysaccharide (LPS), activation markers were followed. Columns represent median with standard error mean from six independent experiments. (**A**)—cell surface presence of CD80; (**B**)—cell surface presence of CD86; (**C**)—cell surface presence of MHCII on BMDC. Representative histograms documenting cell surface presence of activation markers on non-stimulated (blue line), EcO83 HLY+ stimulated (green line) and LPS-stimulated (red line) bone marrow–derived dendritic cells are shown for CD80 (**D**), CD86 (**E**) and MHCII (**F**). Dotted lines stand for histograms of FMO (fluorescence minus one) control. *** *p* ≤ 0.001.

To characterize the impact of probiotic strain *E. coli* O83:K24:H31 (EcO83) on maturation of murine dendritic cells, bone marrow dendritic cells (BMDC) were cocultured with EcO83 for 24 h and activation markers were followed by flow cytometry. Both EcO83 strains (hemolysin positive and negative) were able to promote BMDC maturation to the comparable level with LPS in all markers followed. A significantly increased percentage of activation marker CD80 was observed on CD11c^+^ BMDC after stimulation with EcO83 HLY+ and EcO83- compared with non-stimulated BMDC (*p* ≤ 0.001). Unsurprisingly, LPS was able to increase substantially the presence of CD80 on BMDC (*p* ≤ 0.001); see Figure 1A. Similarly, EcO83 HLY+, EcO83 HLY−, and LPS markedly elevated levels of CD86 on BMDC (*p* ≤ 0.001); see Figure 1B. Both strains of EcO83 and LPS significantly increased the cell surface presence of MHCII on BMDC (*p* ≤ 0.001 for all three treatments); see Figure 1C. Representative histograms for non-stimulated (blue line) and LPS-stimulated (red line) BMDC are shown for CD80 (Figure 1D), CD86 (Figure 1E), and MHCII (Figure 1F).

### 3.2. The Impact of EcO83 on Gene Expression in BMDC and CD4^+^ T Cells Cocultured with Primed BMDC

#### 3.2.1. The Effect of EcO83 on Gene Expression in BMDC In Vitro

**Figure 2:**
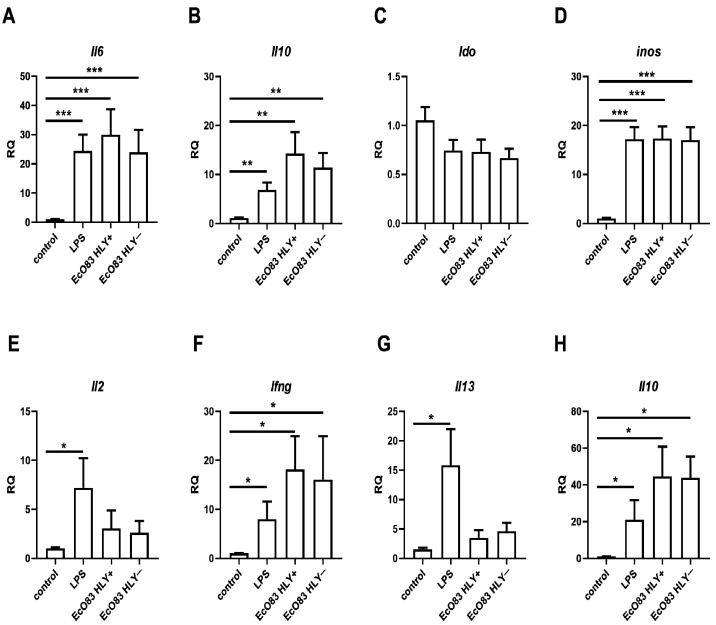
Gene expression in bone marrow-derived dendritic cells and CD4^+^ T cells. Bone marrow dendritic cells were stimulated by hemolysin positive (HLY+) or hemolysin negative (HLY−) strains of *E. coli* O83:K24:H31 (EcO83), with LPS as a positive control. Gene expression was determined by quantitative real-time PCR (qPCR) after 5 h stimulation. Detection of proinflammatory cytokine *Il6*, (**A**), measurement of immunoregulatory markers: *Il10*, (**B**) and indol-amine 2,3 dioxygenase (*Ido*), (**C**). Inducible NO synthase (*inos*) was detected by qPCR, (**D**). Polarization of immune responses after coculture of HLY+ or HLY− *E. coli* O83:K24:H31 (EcO83)-primed bone marrow–derived dendritic cells with naïve CD4^+^ T cells characterized by changes in gene expression of key Th1, Th2, and Treg cytokines. Gene expression of *Il2*—(**E**), gene expression of *Ifng*—(**F**), gene expression of *Il13*—(**G**) and gene expression of *Il10*—(**H**). Columns represent mean with standard error mean from six independent experiments. EcO83 HLY+, hemolysin positive strain of *E. coli* O83:K24:H31; EcO83 HLY−, hemolysin negative strain of *E. coli* O83:K24:H31; RQ, relative quantification of gene expression, * *p* ≤ 0.05, ** *p* ≤ 0.01, *** *p* ≤ 0.001.

To confirm the potential of EcO83 to promote the gene expression of immunoregulatory markers in the BMDC previously observed in human dendritic cells [46], gene expression of selected cytokines, indol-amine 2,3 dioxygenase (*Ido*) and inducible NO synthase [44] was determined by quantitative real-time PCR. Since different cytokines have distinct dynamics of gene expression upon stimulation, the impact of EcO83 on dynamics of gene expression of target genes was determined at various time intervals. Gene expression of pro-inflammatory marker (*Il6*) begins from the early time point intervals (i.e., 30 min and 1 h) reaching the plateau followed by a decrease of gene expression after 5 h; see Supplementary Figure S1A. On the contrary, gene expression of key immunoregulatory cytokine IL-10 is negligible at the early time point intervals compared with later ones. Gene expression of *Il10* remained stable up to 24 h; see Supplementary Figure S1B. No dynamic of gene expression of *Ido* has been observed; see Supplementary Figure S1C. Gene expression of *inos* was steadily increasing even in later time points intervals; see Supplementary Figure S1D. In addition to the dynamics of gene expression of particular genes, the impact of different ratios of bacteria to target cells on gene expression in BMDC has been followed as well. The ratios of 1000, 100, 10 and 1 bacteria to 10 BMDC impacted gene expression of *Il6* significantly compared to non-stimulated control. However, gene expression of *Il6* was lower when ratio of 1 bacteria to 10 BMDC was used compared to higher numbers of bacteria; see Supplementary Figure S2A. Gene expression of *Il10* was concentration dependent while only ratios of 1000, 100 and 10 bacterial cells to 10 BMDC significantly increased gene expression of *Il10*. Ratio of 1 bacteria to 10 BMDC was not sufficient to increase gene expression; see Figure S2B. No ratio of bacteria to BMDC has any impact on gene expression of *Ido*; see Supplementary Figure S2C. Gene expression of *inos* was dependent on ratio of bacteria: BMDC used. The ratios of 1000, 100 and 10 bacteria to 10 BMDC elevated gene expression of *inos* significantly. Increase of gene expression of *inos* when ratio of 1 bacteria to 10 BMDC was used was only marginal and has not reached statistical significance; see Supplementary Figure S2D. Both HLY+ and HLY− strains of EcO83 were able to promote the expression of *Il6*, a typical proinflammatory marker, to levels comparable with the positive control—LPS-stimulated BMDC (*p* ≤ 0.001 for all treatments); see Figure 2A. The capacity of both HLY+ and HLY− strains of EcO83 to induce the gene expression of *Il10* was comparable (*p* = 0.0099 and *p* = 0.0039, respectively). As expected, LPS significantly promoted the gene expression of *Il10* as well (*p* = 0.0016); see Figure 2B. Expression of *Ido* was not impacted by either HLY+ and HLY− EcO83 or by LPS; see Figure 2C. Inducible NO synthase was markedly increased in BMDC-stimulated by HLY+ and HLY− EcO83 and LPS (*p* ≤ 0.001 for all treatments); see Figure 2D.

#### 3.2.2. The Capacity of EcO83-Primed BMDC and Gut DC to Polarize CD4^+^ T Cells

To evaluate the capacity of EcO83-primed BMDC to polarize immune responses, EcO83-stimulated BMDC were cocultured for 7 days with naïve CD4^+^ T cells. LPS stimulation of BMDC significantly increased gene expression of *Il2* in CD4^+^ T cells cocultured with LPS-primed BMDC. The effect of HLY+ and HLY− EcO83 was only marginal and did not reach statistical significance; see Figure 2E. Gene expression of *Ifng* was significantly elevated in CD4^+^ T cells cocultured with LPS, HLY+ and HLY− EcO83-primed BMDC compared to control BMDC cocultured with naïve CD4^+^ T cells. HLY+ and HLY− EcO83 stimulation has comparable effect on gene expression of *Ifng*; see Figure 2F. The effect of EcO83-primed BMDC on changes of gene expression of *Il13* was only marginal. Only LPS-primed BMDC cocultured with naïve CD4^+^ T cells were able to promote gene expression of *Il13*; see Figure 2G. Gene expression of *Il10* was significantly increased in cells cocultured with BMDC primed by LPS and both HLY+ and HLY− strains of EcO83; see Figure 2H.

The capacity of EcO83 primed BMDC to polarize CD4^+^ T cells was assessed based on the presence of intracellular cytokines detected by flow cytometry. Both HLY+ and HLY− strains of EcO83 promoted intracellular presence of IFN-γ to the similar level as LPS; see Supplementary Figure S3A. No effect of EcO83 or LPS primed BMDC on production of Th2 cytokine IL-13 has been observed; see Supplementary Figure S3B. Both HLY+ and HLY− strains of EcO83 increased IL-17A in CD4^+^ T cells compared to both non-stimulated and LPS stimulated BMDC; see Supplementary Figure S3C. BMDC treated by both mutant of EcO83 and LPS promoted intracellular presence of IL-10 in CD4^+^ T cells. When presence of IL-10 in regulatory T cells (Treg) was inspected only HLY+ and HLY− EcO83 stimulated BMDC elevated immunoregulatory cytokine in CD4^+^ T cells compared to non-treated and LPS stimulated BMDC; see Supplementary Figure S3E. 

To compare the capacity of in vitro generated BMDC to polarize immune responses with DC isolated directly from mice, DC from intestine of non-supplemented and HLY+ EcO83 supplemented mice were stimulated with HLY+ EcO83 followed by coculture with CD4^+^ T cells. Priming of DC obtained from both control and EcO83 supplemented mice ex vivo promoted the capacity of DC to induce IFN-γ in CD4^+^ T cells. DC obtained from HLY+ EcO83 treated mice exerted higher potential to promote intracellular presence of IFN-γ compared with DC from naïve mice; see Supplementary Figure S4A. No effect of EcO83 primed DC from both naïve and EcO83 treated mice was reflected in changes of IL-13 secretion; see Supplementary Figure S4B. EcO83 stimulated DC of naïve mice increased IL-17A in CD4^+^ T cells compared to non-stimulated DC of naïve mice. Interestingly, nonstimulated DC from EcO83 treated mice have superior effect on induction of IL-17 secretion as documented by higher intracellular presence of IL-17A. Only nonsignificant increment of IL-17 was observed in CD4^+^ T cells cocultured with EcO83 stimulated DC compared with nonstimulated DC from EcO83 supplemented mice; see Supplementary Figure S4C. EcO83 stimulated DC of naïve mice promoted intracellular presence of IL-10 in CD4^+^ T cells compared to PBS treated DC of naïve mice. PBS treated DC of EcO83 supplemented mice exerted higher potential to induce IL-10 in CD4^+^ T cells compared with PBS treated DC isolated from control mice; see Supplementary Figure S4D. Similarly, EcO83 stimulated DC from naïve mice enhanced intracellular presence of IL-10 in Treg (CD4^+^ FoxP3^+^) compared to non-stimulated DC. PBS treated DC of EcO83 supplemented mice exerted higher potential to induce IL-10 in CD4^+^ T cells compared with PBS treated DC isolated from control mice. However, only minor effect on increment of IL-10 in Treg was observed when EcO83 primed DC of EcO83 supplemented mice were compared with PBS treated DC of EcO83 supplemented mice; see Supplementary Figure S4E. These results are suggesting that DC of EcO83 treated mice have more profound effect on induction of immunoregulatory responses together with IL-17A and IFN-γ production. We have not observed any difference in Treg numbers among particular groups tested (data not provided).

### 3.3. The Effect of EcO83 on Cytokine Production by BMDC and CD4^+^ T Cells Cocultured with Primed BMDC

#### 3.3.1. The Capacity of EcO83 to Modulate Cytokine Production by BMDC

**Figure 3:**
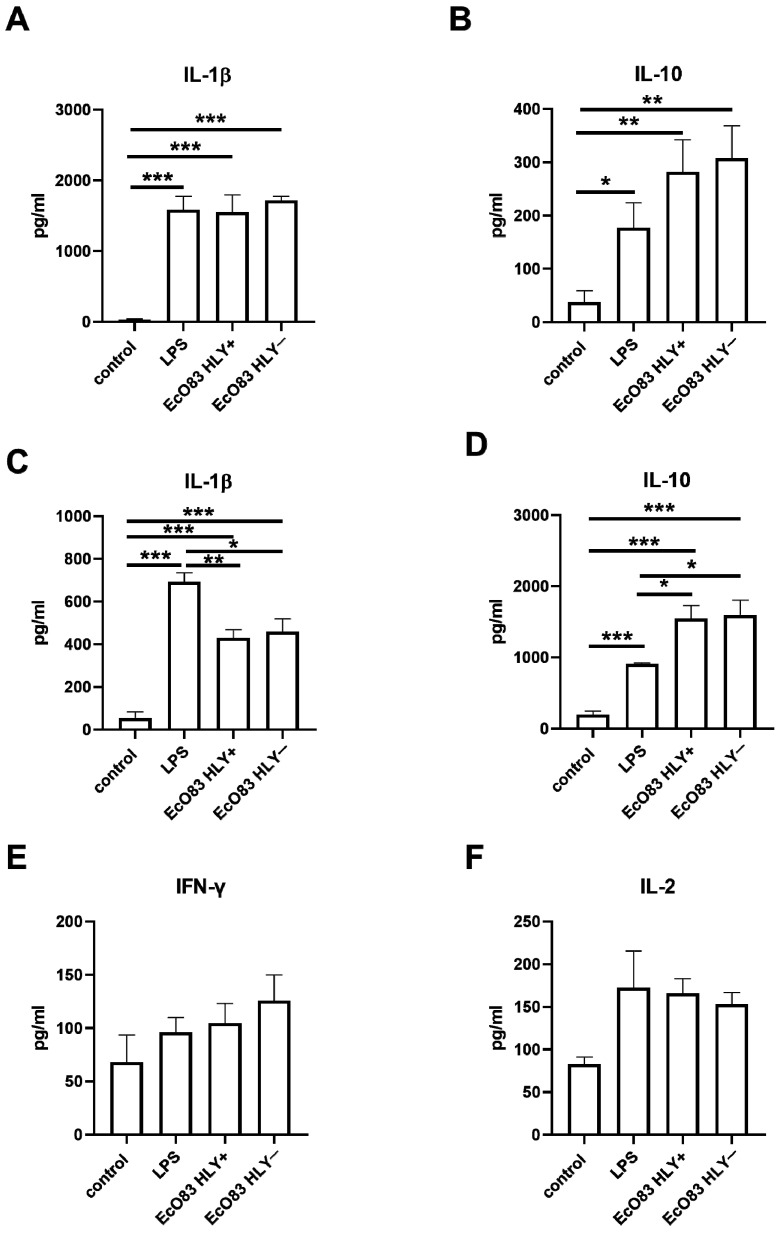
Detection of cytokines in cell culture supernatants. Bone marrow-derived dendritic cells (BMDC) were stimulated by lipopolysaccharide (LPS) and hemolysin positive (HLY+) or hemolysin negative (HLY−) strains of *E. coli* O83:K24:H31 (EcO83) for 24 h and cell culture supernatants were inspected for IL-1β (**A**) and IL-10 (**B**) by ELISA. The capacity of HLY+ and HLY− EcO83- and LPS-stimulated BMDC to modulate cytokine production in naïve CD4^+^ T cells upon coculture. Detection of IL-1β—(**C**), IL-10—(**D**), IFN-γ—(**E**) and IL-2—(**F**) by ELISA. * *p* ≤ 0.05, ** *p* ≤ 0.01, *** *p* ≤ 0.001.

To characterize the effect of EcO83 stimulation on cytokine production, cell culture supernatants of BMDC after 24 h stimulation were inspected for IL-1β, IL-10, and IL-12p70 by ELISA. Concentration of IL-12 in cell culture supernatants was on the border of the detection limit even in LPS- and EcO83-stimulated BMDC. LPS and both HLY+ and HLY− strains of EcO83 significantly increased secretion of IL-1β (in all cases *p* ≤ 0.0001); see Figure 3A. LPS stimulation promoted IL-10 production by BMDC (*p* = 0.0354). Both HLY+ and HLY− EcO83 significantly increased production of IL-10, *p* = 0.0088 and *p* = 0.0058, respectively; see Figure 3B.

#### 3.3.2. The Impact of Primed BMDC on Cytokine Release by CD4^+^ T Cells 

To confirm the immunomodulatory capacity of EcO83 observed on mRNA level, cell culture supernatants of BMDC stimulated by bacteria or LPS were collected after 24 h and cytokines were determined by ELISA. Detection of Th2 cytokines (IL-4 and IL-13) was on the border of the detection limit (data not shown). Coculture of LPS-primed BMDC with naïve CD4^+^ T cells promoted production of IL-1β (*p* ≤ 0.0001). Both HLY+ and HLY− EcO83-primed BMDC increased levels of IL-1β in cell culture supernatants (*p* = 0.0002 and *p* = 0.0010, respectively). Concentrations of IL-1β in cell culture supernatants of HLY+ and HLY− EcO83-stimulated DC cocultured with CD4^+^ T cells were lower compared with LPS-primed BMDC (*p* = 0.0040 and *p* = 0.0198, respectively); see Figure 3C. LPS and EcO83-stimulated BMDC cocultured with naïve CD4^+^ T cells promoted IL-10 secretion (*p* ≤ 0.0001 in all cases) but both HLY+ and HLY− EcO83 stimulation was superior in IL-10 production compared with LPS stimulation (*p* = 0.0143 and *p* = 0.0178, respectively); see Figure 3D. The capacity of LPS and EcO83-stimulated BMDC to promote IFN-γ gene expression in CD4^+^ T cells was not confirmed on protein level. Only minor non-significant trend to increased levels of IFN-γ in cell culture supernatants was observed; see Figure 3E. Similarly, only marginal effect of LPS and EcO83-stimulated BMDC on secretion of IL-2 by CD4^+^ T cells detected; see Figure 3F.

### 3.4. Persistence of EcO83 in Neonatal Gastrointestinal Tract

**Figure 4:**
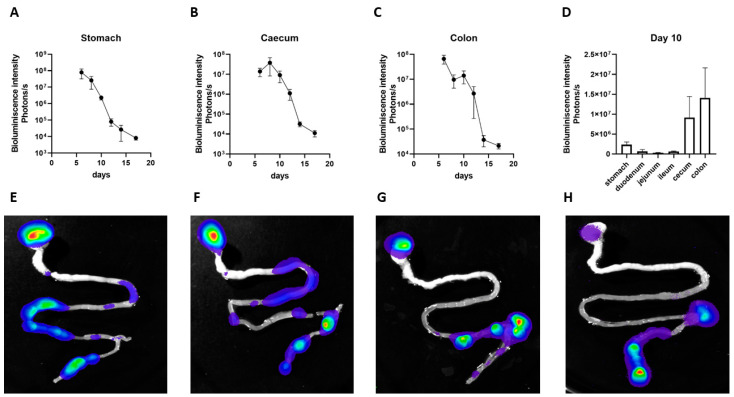
Tracking of colonization capacity of *E. coli* O83:K24:H31 (EcO83) in particular parts of gastrointestinal tract of neonatal mice. Mice were colonized by transfected strain of EcO83 expressing luciferase and the presence of EcO83 in gastrointestinal tract of neonatal mice was followed by measurement of bioluminescence activity using bioimaging. The dynamics of bioluminescence intensity is shown for stomach (**A**), caecum (**B**), and colon (**C**). The difference in presence of EcO83 among particular parts of gastrointestinal tract measured by bioluminescence intensity at day 10 is shown in (**D**). Each time point represents mean with standard error mean from three mice from one representative experiment. Representative photos documenting the dynamics of colonization capacity of EcO83 are shown in (**E**–**H**).

Early postnatal probiotic supplementation has a great potential to affect immune system maturation and to induce appropriate tolerogenic responses. To evaluate the persistence of EcO83-expressing luciferase in the neonatal gastrointestinal tract of mice, EcO83 was administered within 24 h after delivery and luciferase activity was followed. The highest bioluminescence was observed in the stomach in the early time points after oral application of EcO83. The dynamics of the colonization persistence of EcO83 in individual parts of gastrointestinal tract of neonatal mice is documented in Figure 4. The bioluminescence moved to distal part of the gastrointestinal tract of the neonatal mice, but the signal coming from stomach was higher compared to the small intestine at day 10; see Figure 4D. The representative figures documenting the dynamics of the persistence of EcO83 in the neonatal gut are shown in Figure 4E–H. This phenomenon of EcO83 persistence in the stomach was not observed when adult mice were supplemented with EcO83.

### 3.5. Effect of Early Postantal EcO83 on Cytokine Expression in Gut

**Figure 5:**
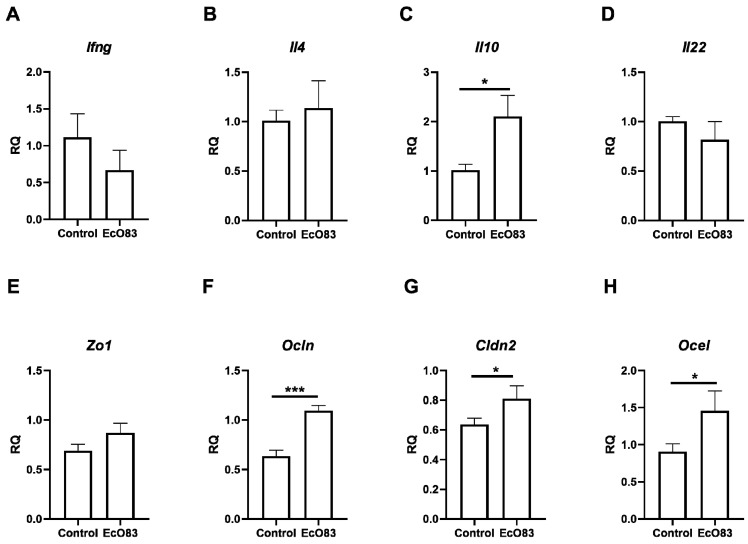
The impact of early postnatal *E. coli* O83:K24:H31 (EcO83) administration on gene expression of cytokines and tight junction proteins in neonatal gut measured by quantitative real-time PCR at the age of 10 days. (**A**)—gene expression of *Ifng*; (**B**)—gene expression of *Il4*; (**C**)—gene expression of *Il10*; (**D**)—gene expression of *Il22.* Gene expression of proteins associated with barrier function: expression of *zonulin 1* (*Zo1*)—(**E**); expression of *occludin* (*Ocln*)—(**F**); expression of *claudin 2* (*Cldn2*)—(**G**); and expression of *occludin/ELL domain containing 1* (*Ocel*)—(**H**). One representative experiment of three independent experiments is shown. Columns represent mean with standard error mean of 12 mice in total. * *p* ≤ 0.05, *** *p* ≤ 0.001.

The impact of early postnatal oral EcO83 administration on the maturation of the neonatal immune system and gut barrier function was followed. To confirm the capacity of EcO83 to promote immunoregulatory function, the gene expression of pro-inflammatory cytokine *Il10* and other cytokines promoting Th1 (*Ifng*), Th2 (*Il4*) and gut homeostasis (*Il22*) was measured by quantitative real-time PCR in the ileum. We did not observe any effects of EcO83 application on changes in gene expression of typical Th1 (*Ifng*) and Th2 (*Il4*) cytokines; see Figure 5A,B, respectively. Early postnatal administration of EcO83 promoted the expression of key immunoregulatory cytokine *Il10* (*p* = 0.0347); see Figure 5C. Nevertheless, early postnatal EcO83 application had no effect on the gene expression of *Il22* in the neonatal gut; see Figure 5C. 

### 3.6. The Effect of Early Postnatal Administration of EcO83 on Gut Barrier Functions

To determine whether early postnatal EcO83 supplementation promoted gut barrier function, the gene expression of tight junction proteins was measured by quantitative real-time PCR. Only a non-significant trend of increased gene expression of *zonulin 1* was observed in EcO83-treated mice; see Figure 5E. Early postnatal EcO83 supplementation significantly promoted gene expression of *occludin* (*p* = 0.0004; Figure 5F), *claudin 2* (*p* = 0.0361; Figure 5G), and *occludin/ELL domain containing 1* (*p* = 0.0213; Figure 5H).

### 3.7. The Impact of Early Postnatal EcO83 Administration on Selected Cellular Subsets in Gut

To evaluate the impact of early postnatal EcO83 administration on the proportion and functional capacity of immune cells in the mesenteric lymphoid nodes (MLN) and the gut, selected markers were traced by flow cytometry. We did not observe significant effects of EcO83 supplementation on the proportion of dendritic cells (DC) in the MLN and gut (data not shown). The impact of EcO83 supplementation on the presence of cell surface activation markers was only marginal and did not reach statistical significance; see Supplementary Figure S5. Similarly, the proportions of Treg (CD4^+^ CD25^+^) and CD4^+^ CCR7^+^ were not significantly impacted by early postnatal EcO83 application (Supplementary Figure S6). It seems that the functional capacities were impacted rather than the proportion of Treg itself.

## 4. Discussion

The capacity of *Escherichia coli* O83:K24:H31 (EcO83) with and without hemolysin to modulate immune responses was compared. Both strains of EcO83 exerted comparable immunomodulatory capacity including induction of dendritic cell maturation and the expression of cytokines. Importantly, coculture of HLY+ and HLY− EcO83-primed BMDC with naïve CD4^+^ T cells was able to promote *Ifng* and *Il10* expression, highlighting the potential of EcO83 to induce the maturation of the neonatal immune system and induce appropriate immunoregulatory function. In addition to that, early postnatal EcO83 application was able to promote gut barrier function and immunoregulatory capacity.

The key role of microbiota on the maturation of the neonatal immune system was acknowledged. Children born by Caesarean section have different microbiota compositions affecting the maturation of the immune system, possibly predisposing these individuals to immune-mediated diseases (e.g., IBD, allergies, autoimmune diseases) [7]. Supplementation of newborns with suitable probiotics could prevent dysbiosis and promote immune system maturation [17,18,47,48]. Several studies reported early postnatal probiotic supplementation lowered incidence of immune-mediated diseases [36,48,49,50]. Unfortunately, knowledge about the mechanisms of how the probiotics impact maturation of the neonatal immune system is scarce. We have to consider the safety of probiotics as well. The beneficial effect of *Escherichia coli* O83:K24:H31 (EcO83) in the prevention of allergy development has been reported, but this particular strain has low hemolysin activity [39]. Therefore, strains without hemolytic activity would represent the safer option. The immunomodulatory capacity of HLY+ and HLY− EcO83 were comparable, as seen in Figure 1, Figure 2 and Figure 3. It is important to emphasize the *E. coli* has immunodominant antigen LPS. Since both mutants, HLY+ and HLY−, have the same immunodominant antigen, it is possible to hypothesize that the effect of the presence/absence of hemolysin on changes of immune responses cannot be detected using our experimental methodological approaches. Both strains were able to promote the maturation of dendritic cells while preserving their capacity to trigger the expression and production of the key immunoregulatory cytokine IL-10. Importantly, the ratio of bacteria to target cells used can impact the results as suggested in Figure S2. In addition to that, dynamics of gene expression of particular cytokines differs among cytokines and has to be considered as demonstrated in Supplementary Figure S1. This finding confirms our previous observation, where dendritic cells generated from cord blood precursors were used [46]. In this paper, EcO83 was able to promote expression of IL-10 and IDO. To our surprise, IDO expression in murine DC (BMDC) was not affected by LPS or by EcO83 stimulation. The necessity of two signals to reach sufficient IDO expression in DC, reviewed by Harden et al. [51], could explain our observation of missing increments in the gene expression of IDO in LPS- or EcO83-stimulated BMDC. Nevertheless, only one signal (LPS or EcO83 stimulation) was sufficient to induce the gene expression of IDO using human DC in the previous study [46]. Similarly, probiotic bacteria were able to promote IDO expression in DC obtained from healthy individuals and patients suffering from systemic lupus erythematosus [52]. This observation of the different responses of human and mouse DC to the same stimuli highlights the difference between the human and murine immune systems, which has to be considered when observations from preclinical animal models are transferred into the design of human clinical trials.

It seems that the propensity of probiotics to promote IL-10 expression or production is a critical characteristic of bacterial strains capable of preventing or downregulating inflammatory disorders. Importantly, the amelioration of the severity of experimental colitis was IL-10 dependent [53,54,55]. Lowering the clinical degree of experimental arthritis by lactobacilli administration was IL-10 dependent as well [56]. Similarly, the capacity of lactobacilli to induce oral tolerance via Treg induction can be exploited in the prevention of food allergies [57] or in lowering the severity of experimental autoimmune encephalomyelitis [58]. We observed the capacity of EcO83 to induce IL-10 in both BMDC and T cells. Similarly, the probiotic mixture VSL#3 was demonstrated to promote IL-10 production by DC [59,60]. Interestingly, EcO83 was not able to dampen the expression of pro-inflammatory markers *Il6* and *inos*. The impaired capacity of EcO83 to limit pro-inflammatory markers was observed in human DC as well [46]. On the other hand, several other probiotics have been described as having the potential to downregulate the expression of iNOS [61]. This emphasizes that different probiotic strains exert different immunomodulatory potentials, highlighting the importance of the selection of an appropriate probiotic strain depending on the desired effect on the immune system and the importance of taking into account the high interindividual differences among people.

Dendritic cells, as professional antigen presenting cells, play a central role in the priming/polarization of adaptive immune responses. Therefore, the capacity of probiotics to modulate the functional characteristics of DC could be reflected by changes in proportion of the effector subsets of Th1/Th2/Th17/Treg. We observed increased *Il10* expression after coculture of naïve CD4^+^ T cells with EcO83-primed BMDC, confirming the strong immunoregulatory effect of EcO83, which could be exploited in the dampening of undesirable pro-inflammatory responses [62]. The neonatal immune system is generally immature with the predominance of Th2 immune responses [63]. Therefore, the capacity of probiotic strains to promote the maturation of the immune system by balancing Th1 and Th2 immune responses and inducing appropriate immunoregulatory function would be optimal. EcO83 exerted the potential to promote the Th1 immune response, together with elevating IL-10 expression and production. Nevertheless, EcO83 was not able to limit Th2 immune responses. This observation is in agreement with previously published work using human cord blood mononuclear cells [36]. Importantly, the promotion of Th1 immune responses was described for other probiotic strains suitable for the treatment of allergic diseases [64].

Probiotic strains can have direct effects on the immune system or the effects can be mediated via the modification of the microbiota composition or metabolite production [65]. EcO83 has a good colonization capacity and its continuous presence was confirmed one year after the primary colonization in newborns [66]. In the current study, the presence of EcO83 was tracked by bioimaging in neonatal mice. The high abundance of EcO83 in the gastrointestinal tract decreased by day 15, possibly as other microorganisms became dominant in the developing gut microbiota of the pups. Nevertheless, this strong presence of EcO83 during the early postnatal period could have huge and possibly long-term effects on inducing immune responses in neonates. To our surprise, higher bioluminescence of EcO83 was observed in the stomachs of neonatal mice after oral application of EcO83 compared to adults (data not shown). This can be due to the lower gastric secretion and lower gastric pH in neonates compared with adults. The colonization capacity of another probiotic strain, *E. coli* Nissle 1917, was reported and an exclusive colonization of the gut was observed in adult mice [67].

The early postnatal EcO83 administration promoted gut barrier function together with immunoregulatory capacity. Barrier function seems to be critical in preventing inflammatory bowel disease development [68,69]. Bacteria have been described as promoting barrier function, leading to the amelioration of colitis [70,71]. Importantly, tight junction protein expression was altered in newborns predisposed to the development of necrotizing enterocolitis [72]. Moreover, neonates do not have fully developed tight junctions; therefore, the promotion of the development of barrier function is critical. Neonates after antibiotic treatment are more prone to developing necrotizing enterocolitis, so these individuals may also benefit from probiotic supplementation [73].

Early postnatal supplementation can have a higher impact on host health for several reasons. Firstly, newborns have only limited microbial colonization; therefore, probiotics administered early postnatally do not compete for nutrients with fully developed microbiota. Secondly, the immune system is immature, and providing probiotics has a more profound effect on shaping the immature immune system compared to the restoration of altered immune system responses in adulthood. Additional experiments employing distinct experimental models of human diseases caused by impaired immunoregulation should be performed to clarify the clinical relevance of suggested modes of action of EcO83. Only moderate protection of EcO83 has been demonstrated in dextran sodium sulfate-induced colitis challenging the immunoregulatory capacity of EcO83 [74]. It has been proposed that one of the factors pointing to protective capacity of particular probiotic strain in colitis is the endotoxicity of its LPS [75]. Nevertheless, further studies are needed to clarify the long-term effects of early probiotic administration on both the host immune system and microbiota composition and function in adulthood.

## Figures and Tables

**Table 1:** List of TaqMan gene expression assays.

Gene Name (Abbreviation)	Catatogue Number of TaqMan Gene Expression Assay
Beta-actin (*Actb*)	Mm00607939_s1
Interleukin 2 (*Il2*)	Mm00434256_m1
Interleukin 4 (*Il4*)	Mm99999154_m1
Interleukin 6 (*Il6*)	Mm99999064_m1
Interleukin 10 (*Il10*)	Mm00439616_m1
Interleukin 13 (*Il13*)	Mm99999190_m1
Interleukin 22 (*Il22*)	Mm00444241_m1
Interferon-gamma (*Ifng*)	Mm00801778_m1
Inducible NO synthase (*inos*)	Mm00440485_m1
Indol amine 2,3 dioxygenase (*Ido*)	Mm00492590_m1
occludin/ELL domain containing 1 (*Ocel*)	Mm01349279_m1
Zonulin 1 (*Tjp1, Zo1*)	Mm00493699_m1
Occludin (*Ocln*)	Mm00500912_m1
Claudin 2 (*Cldn2*)	Mm00516703_s1

## Data Availability

Data are available upon reasonable request from corresponding author.

## Supplementary Materials

The following supporting information can be downloaded at: https://www.mdpi.com/article/10.3390/cells11101624/s1. Figure S1. The effect of the duration of stimulation on gene expression of target genes, Figure S2. The effect of different bacteria:cell ratios on gene expression of target genes in bone marrow-derived dendritic cells, Figure S3. The capacity of hemolysin positive (HLY+) and hemolysin negative (HLY−) strains of *Escherichia coli* O83:K24:H31 (EcO83) primed bone marrow-derived dendritic cells (BMDC) to induce particular subsets of CD4^+^ T cells detected by flow cytometry, Figure S4. The capacity of hemolysin positive (HLY+) *Escherichia coli* O83:K24:H31 (EcO83) primed dendritic cells (DC) isolated from mouse intestine to polarize particular subpopulations of CD4^+^ T cells, Figure S5. The impact of early postnatal administration of probiotic strain *Escherichia coli* O83:K24:H31 (EcO83) on maturation status of dendritic cells determined by flow cytometry, Figure S6. The impact of early postnatal EcO83 administration on T cells.
