# Supplementary material for: The Impact of Escherichia coli Probiotic Strain O83:K24:H31 on the Maturation of Dendritic Cells and Immunoregulatory Functions In Vitro and In Vivo
Source: Cells. 2022 May 12;11(10):1624. doi: 10.3390/cells11101624 (PMC9140140; doi:10.3390/cells11101624)
Supplement: Supplementary file 1 [file cells-11-01624-s001.zip › cells-1590177-supplementary.pdf]

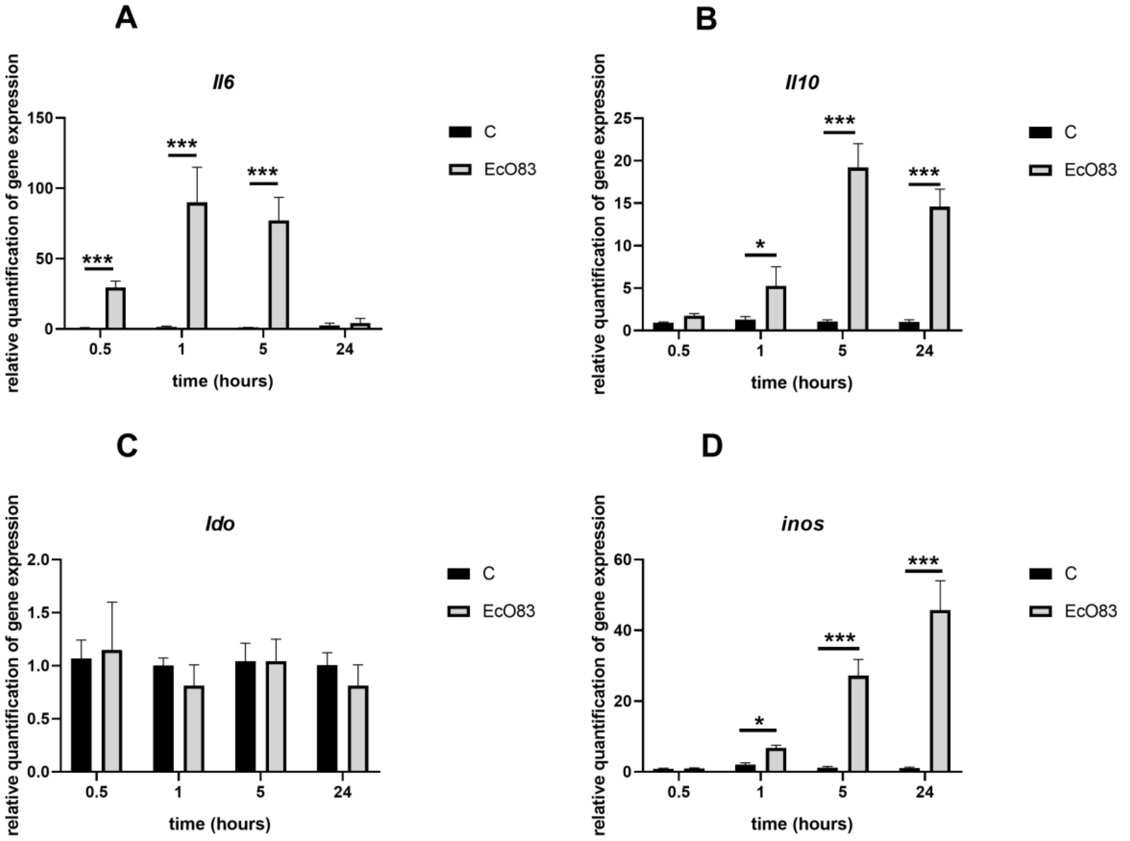


**Figure S1.** The effect of the duration of stimulation on gene expression of target genes. Bone marrow-derived dendritic cells were stimulated with *Escherichia coli* O83:K24:H31 for 0.5; 1; 5 and 24 hrs followed by measurement of gene expression of *Il6* (**A**), *Il10* (**B**), indol amine 2,3 dioxygenase (*Ido*) (**C**) and inducible NO synthase (*inos*) (**D**) by quantitative real-time PCR. Columns represent mean and standard error mean of 5 independent experiments. * $p \leq 0.05$, *** $p \leq 0.001$.
C – non-stimulated control bone marrow-derived dendritic cells (BMDC);
EcO83 – BMDC stimulated with *Escherichia coli* O83:K24:H31 in the ratio 10:1 (i.e. 10 bacterial cells:1 BMDC).

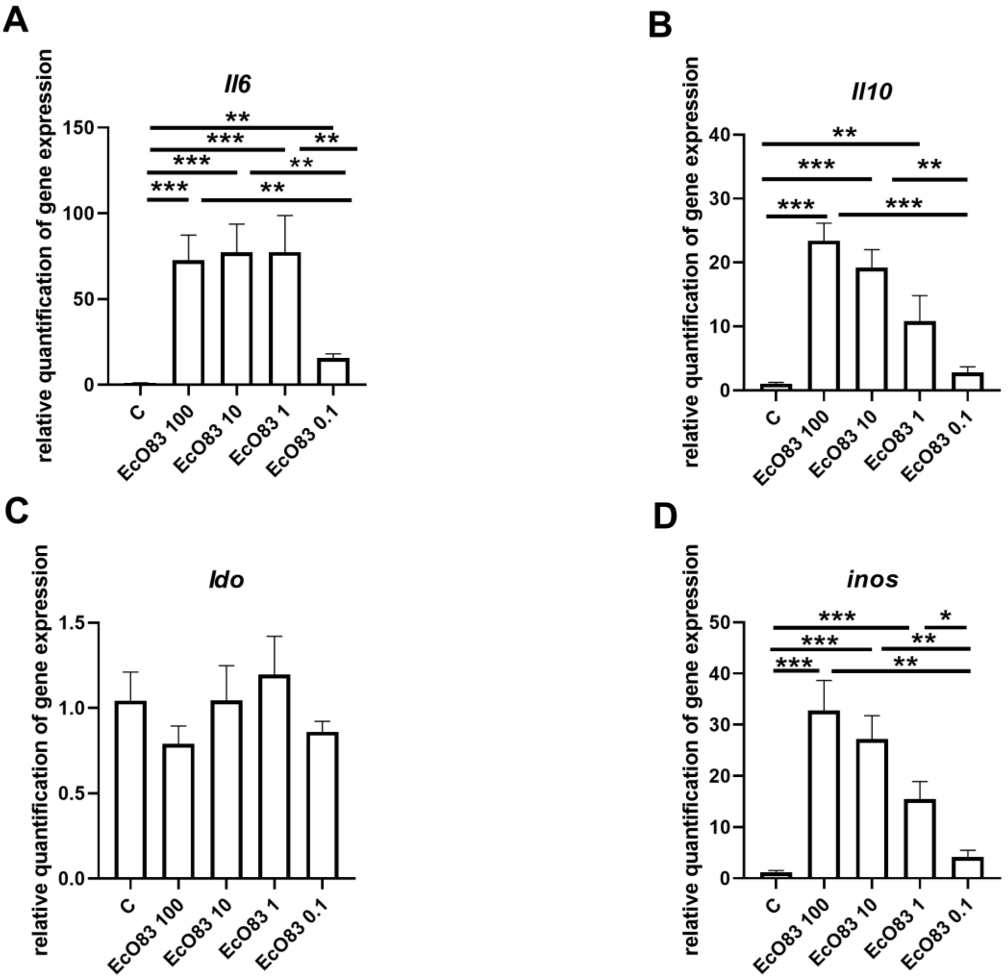


**Figure S2.** The effect of different bacteria : cell ratios on gene expression of target genes in bone marrow-derived dendritic cells. Gene expression of *Il6* (**A**), *Il10* (**B**), indol amine 2,3 dioxygenase (*Ido*) (**C**) and inducible NO synthase (*inos*) (**D**) was determined after 5 h stimulation. Columns represent mean and standard error mean of 5 independent experiments. * $p \leq 0.05$, ** $p \leq 0.01$, *** $p \leq 0.001$.

C – non-stimulated control bone marrow-derived dendritic cells (BMDC); EcO83 100 – BMDC stimulated with *Escherichia coli* O83:K24:H31 in the ratio 1:100 (i.e. 1 BMDC : 100 bacterial cells);

EcO83 10 – BMDC stimulated with *Escherichia coli* O83:K24:H31 in the ratio 1:10 (i.e. 1 BMDC : 10 bacterial cells);

EcO83 1 – BMDC stimulated with *Escherichia coli* O83:K24:H31 in the ratio 1:1 (i.e. 1 BMDC : 1 bacterial cell);

EcO83 0.1 – BMDC stimulated with *Escherichia coli* O83:K24:H31 in the ratio 10:1 (i.e. 10 BMDC:1 bacterial cell)

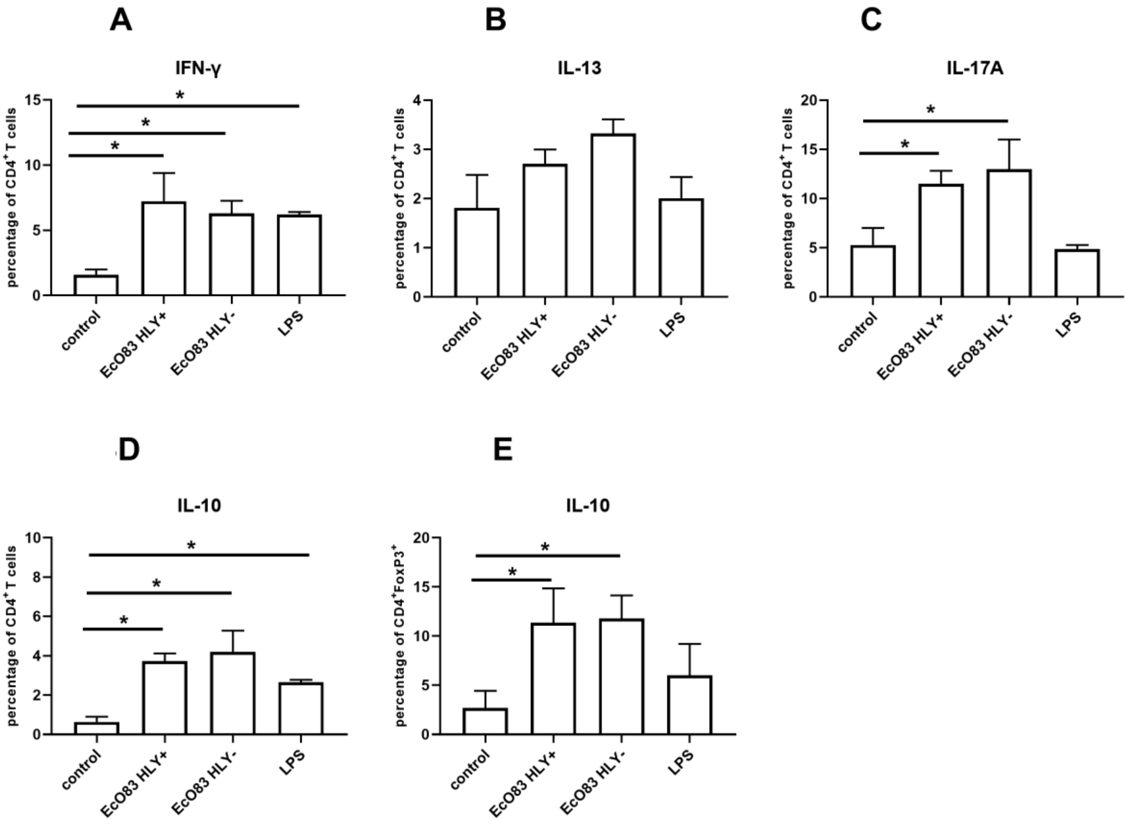


**Figure S3.** The capacity of hemolysin positive (HLY+) and hemolysin negative (HLY-) strains of *Escherichia coli* O83:K24:H31 (EcO83) primed bone marrow-derived dendritic cells (BMDC) to induce particular subsets of $CD4^{+}$T cells detected by flow cytometry. Intracellular presence of IFN-$\gamma$, (**A**), IL-13 (**B**), IL-17A (**C**) and IL-10 (**D**) in $CD4^{+}$T cells was detected by flow cytometry. The capacity of EcO83 to promote IL-10 in regulatory T cells ($CD4^{+}FoxP3^{+}$ cells) was evaluated as well, (**E**) Columns represent mean and standard error mean of 3 independent experiments. * $p \leq 0.05$.

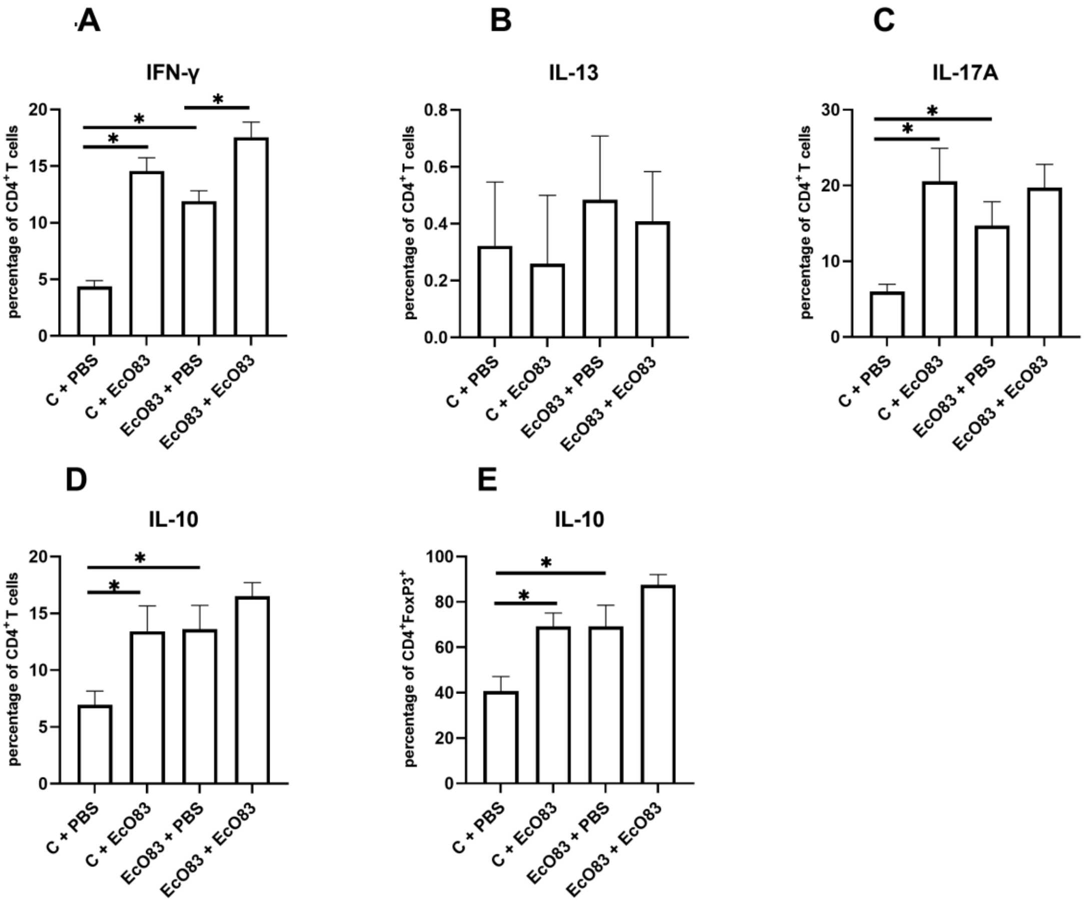


**Figure S4**. The capacity of hemolysin positive (HLY+) *Escherichia coli* O83:K24:H31 (EcO83) primed dendritic cells (DC) isolated from mouse intestine to polarize particular subpopulations of $CD4^{+}T$ cells. Intracellular presence of IFN-γ, (**A**), IL-13 (**B**), IL-17A (**C**) and IL-10 (**D**) in $CD4^{+}T$ cells was detected by flow cytometry. The impact of EcO83 primed DC on functional capacity of regulatory T cells was estimated according to the intracellular presence of IL-10, (**E**) Columns represent mean and standard error mean of 3 independent experiments. * $p \leq 0.05$

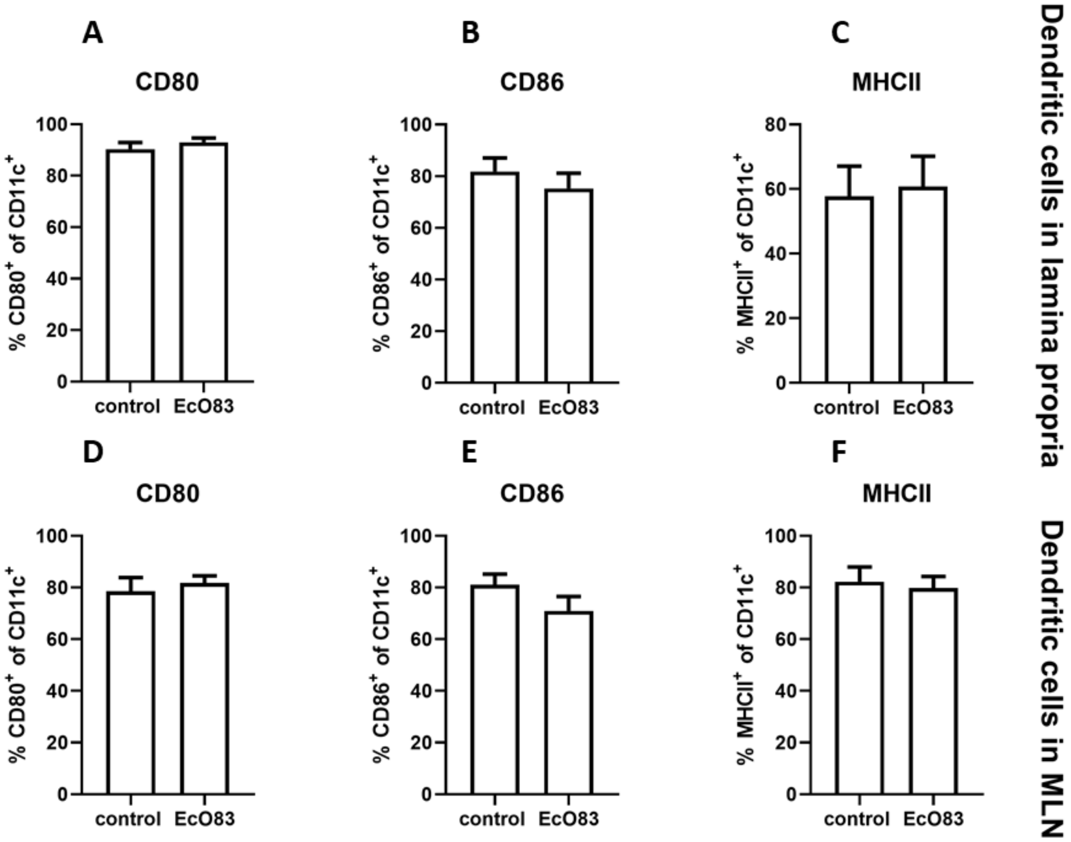


**Figure S5**. The impact of early postnatal administration of probiotic strain *Escherichia coli* O83:K24:H31 (EcO83) on maturation status of dendritic cells determined by flow cytometry. Cell surface presence of activation markers on dendritic cells in lamina propria (**A**–**C**) and mesenteric lymph node (MLN) (**D**–**F**). Columns represent mean with standard error mean from three independent experiments (4 mice per group per experiment).

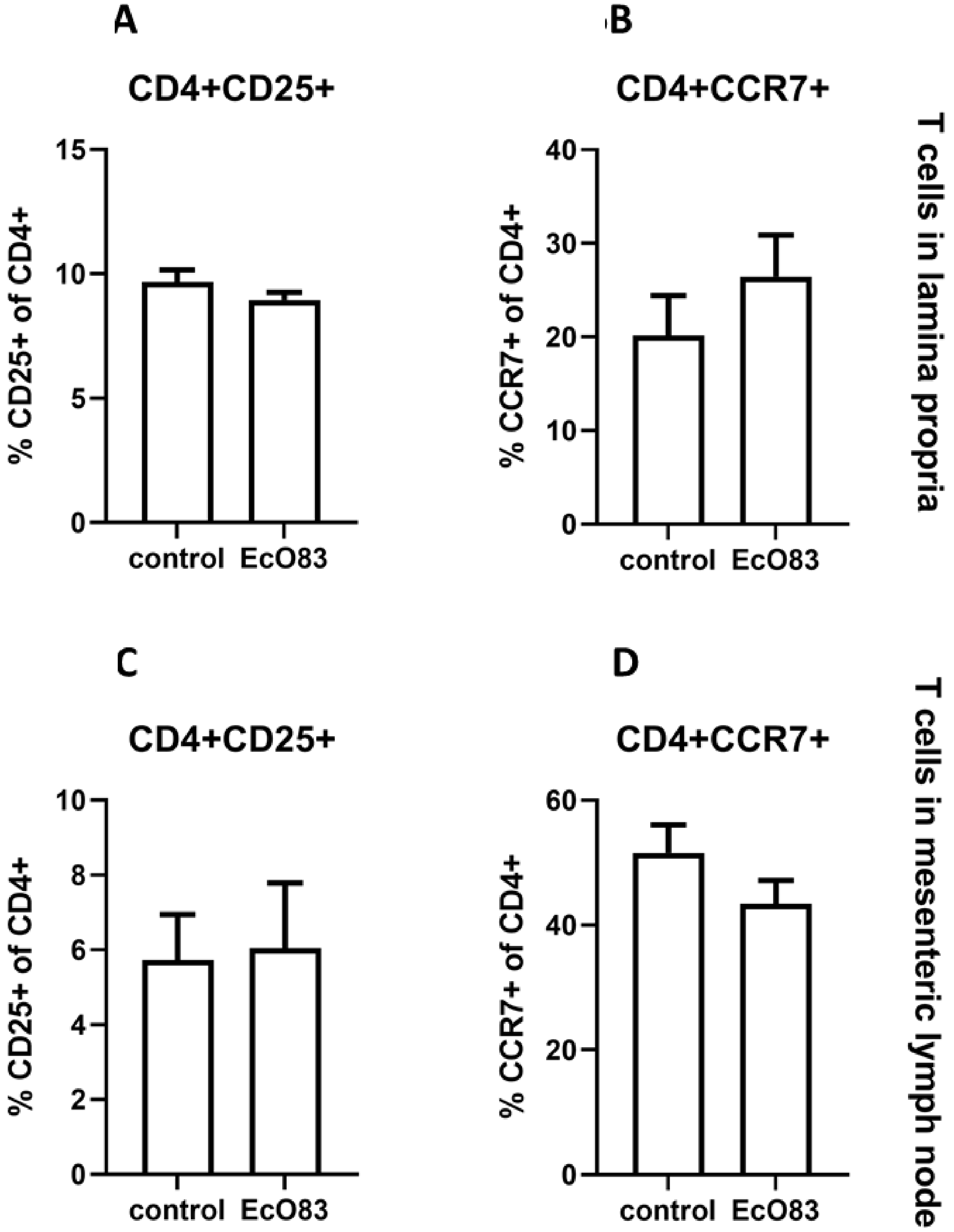


**Figure S6.** The impact of early postnatal EcO83 administration on T cells. Changes in proportion of Treg ($CD4^+CD25^+$) and $CCR7^+$ T cells in gut lamina propria of control and EcO83 treated mice, (**A**) and (**B**), respectively. Proportion of Treg and $CCR7^+$ T cells in mesenteric lymph node of control and EcO83 supplemented mice, (**C**) and (**D**), respectively.
